# Invisible light inside the natural blind spot alters brightness at a remote location

**DOI:** 10.1038/s41598-018-25920-9

**Published:** 2018-05-15

**Authors:** Marina Saito, Kentaro Miyamoto, Yusuke Uchiyama, Ikuya Murakami

**Affiliations:** 1 Department of Psychology, The University of Tokyo, Tokyo, 113-0033 Japan; 2 Department of Physiology, The University of Tokyo School of Medicine, Tokyo, 113-0033 Japan; 3 Department of Experimental Psychology, University of Oxford, Oxford, OX1 3UD United Kingdom; 4 Japan Society for the Promotion of Science, Tokyo, 102-8472 Japan

## Abstract

The natural blind spot in the visual field has been known as a large oval region that cannot receive any optical input because it corresponds to the retinal optic disk containing no rod/cone-photoreceptors. Recently, stimulation inside the blind spot was found to enhance, but not trigger, the pupillary light reflex. However, it is unknown whether blind-spot stimulation also affects visual perception. We addressed this question using psychophysical brightness-matching experiments. We found that a test stimulus outside the blind spot was judged as darker when it was accompanied by a consciously unexperienced blue oval inside the blind spot; moreover, the pupillary light reflex was enhanced. These findings suggested that a photo-sensitive mechanism inside the optic disk, presumably involving the photopigment melanopsin, contributes to our image-forming vision and provides a ‘reference’ for calibrating the perceived brightness of visual objects.

## introduction

The blind spot (BS)^1–4^ corresponds to the optic disk on the retina, where blood vessels and ganglion-cell axons converge to form the optic nerve, which leads away from the eyeball to the brain. For this reason, the optic disk contains no photoreceptors (rods or cones), and thus no visual events can be received within the blind spot. To compensate for the lost visual information, our visual system has a mechanism for perceptual filling-in^5,6^, which creates the visual scene inside the BS by referencing the surrounding information. Hence, we experience a seamless visual world. Recently, we found that, even though the BS has no rods/cones, the involuntary pupil reflex—known as the short-latency pupillary light reflex (PLR)^7,8^—in response to light increments outside the BS was enhanced by concurrent light exposure within the BS^8^. This enhancement was not related to perceptual filling-in; instead, direct light stimulation inside the BS was the key factor. Moreover, the enhancement was more marked when the BS was illuminated by blue rather than red light, suggesting that the mechanism is more sensitive to shorter wavelengths. However, it is unknown whether and how incident light within the optic disk influences our perceptual judgement.

## Results

### Blind-spot illumination causes darkening of a visual stimulus outside the blind spot

**Figure 1:**
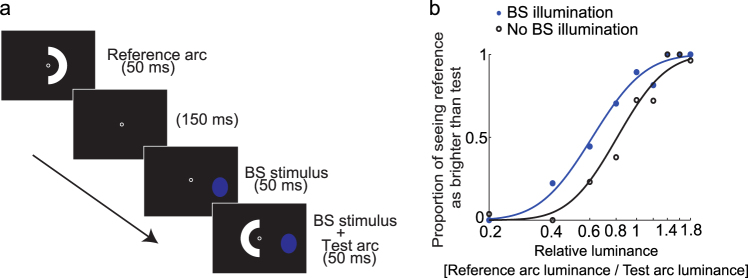
Procedure of Experiment 1 to determine brightness with and without the illumination of the blind spot (BS). (**a**) Brightness-matching task. Observers were asked to judge whether the test arc was brighter or darker than the reference arc. In half of the trials, a blue oval was presented within the blind spot when the test arc appeared. (**b**) Psychometric functions of a representative observer. The proportion of trials in which the reference arc appeared brighter than the test arc is plotted as a function of relative luminance (reference arc luminance/test arc luminance), with (blue) and without (black) BS illumination. Note that the function of the ‘No BS illumination’ condition was not necessarily centred at 1 along the abscissa. This was because the experiment allowed a constant error to originate from the fixed spatiotemporal setup, wherein the reference arc was first presented in the right hemifield, followed by the test arc in the left hemifield. Under BS illumination, the psychometric function shifted leftward from the control condition, indicating that the test arc had become *darker*.

**Figure 2:**
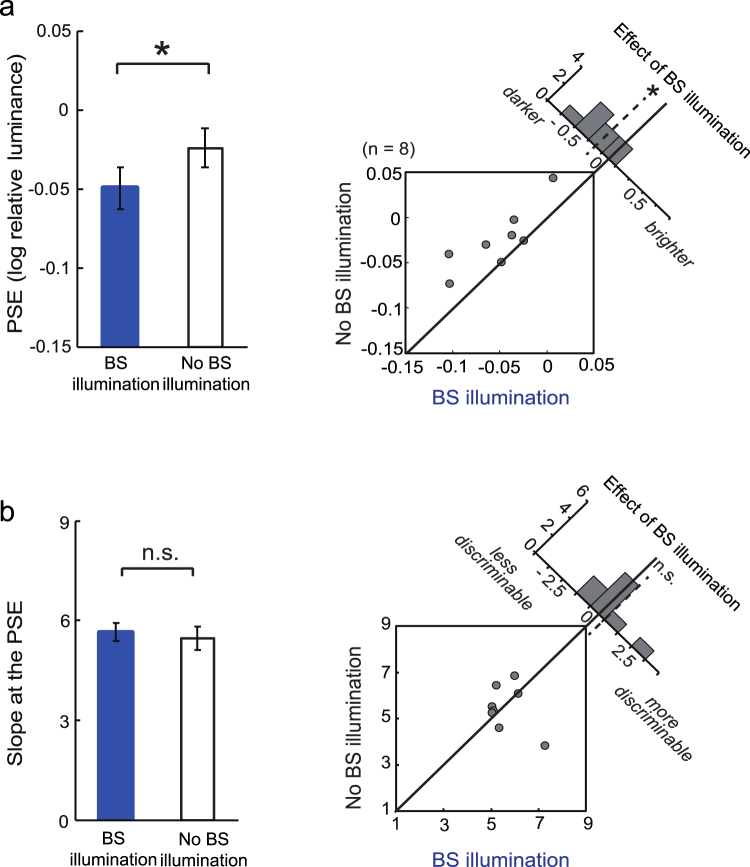
Results of Experiment 1 demonstrating brightness decrease by illumination inside the blind spot (BS). (**a**) Results of the brightness matching. Point of subjective equality (PSE) (n = 8; mean ± standard error) is plotted as an index of brightness in the two conditions. The right-hand panel depicts an interobserver scattergram, as well as a histogram of the difference in PSE between the two conditions; negative values indicate that the test arc appeared darker with BS illumination than without it. *p < 0.05. (**b**) Slope of the psychometric function plotted for the two conditions. The right-hand panel shows an interobserver scattergram, as well as a histogram following the same convention as in (**a)**. n.s.: not significant.

To examine whether light stimulation inside the BS affects the brightness of a visual stimulus elsewhere in the visual field, we conducted psychophysical brightness-matching experiments. In Experiment 1, two white arcs, were sequentially flashed (Fig. 1a). Observers were asked to judge whether the second arc (‘test’) was brighter or darker than the first arc (‘reference’). In half of the trials, the test arc was accompanied by a blue oval *inside* the BS, hereafter called the ‘BS illumination’, whereas the reference arc was never accompanied by BS illumination. We examined whether BS illumination affected the point of subjective equality (PSE) in brightness, i.e., the luminance of the reference arc that appeared just as bright as the test arc (Fig. 1b). Paradoxically, the test arc was judged as *darker* (*t*_7_ = −3.41, *p* = 0.0113) with the BS illumination than without it (Fig. 2a). In contrast, the slope of psychometric function remained similar regardless of BS illumination (*t*_7_ = −1.26, *p* = 0.248; Fig. 2b). Thus, BS illumination shifted brightness without influencing discriminability. The test arc was always presented after the reference arc, to ensure that any sluggish effects caused by BS illumination could not have influenced the results. The test arc disappeared 300 ms after the onset of the reference arc—much earlier than the PLR latency (mean ± standard error: 422 ± 30.6 ms), so pupil constriction could not have influenced the brightness judgement.

### Local light misalignment or scatter outside the blind spot does not account for the darkening effect

**Figure 3:**
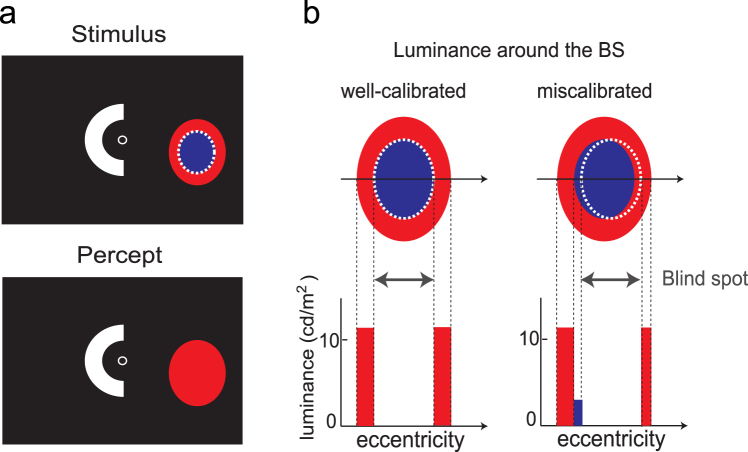
Stimuli used in Experiment 2 examining whether the darkening effect is due to light leakage outside the blind spot (BS). (**a**) Stimulus configuration. A red annulus surrounding the blue BS illumination was presented simultaneously with the test and reference arcs. (**b**) Schematic of the rationale for this control. If BS illumination was not properly confined within the BS, the total luminance available to the visual system would decrease.

**Figure 4:**
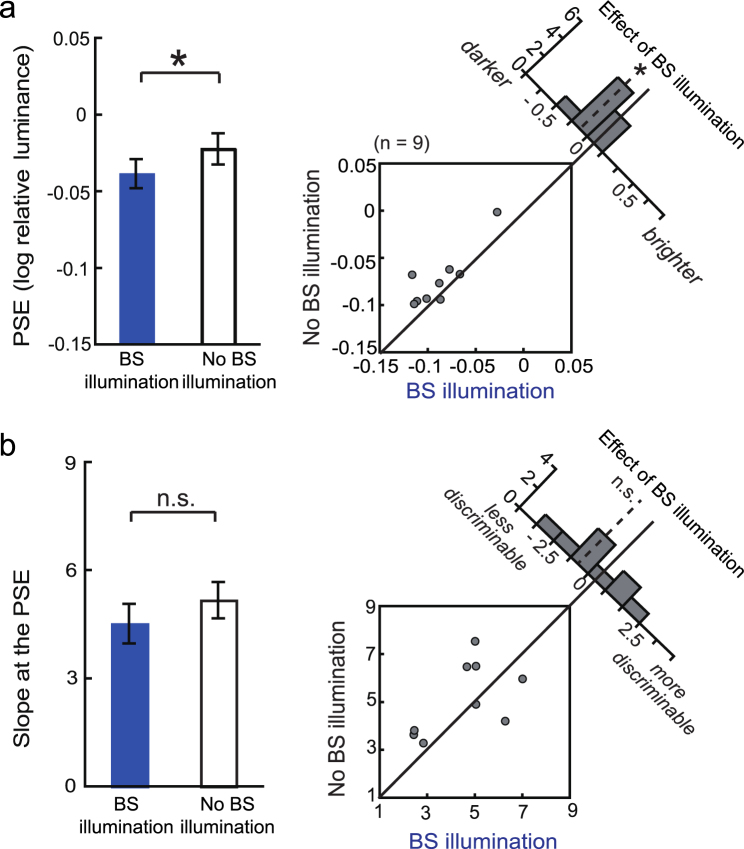
Results of Experiment 2 confirming brightness decrease by the blind-spot illumination despite the control of light leakage. (**a**) Point of subjective equality (n = 10; mean ± standard error). Conventions are the same as those in Fig. 2a. *p < 0.05. (**b**) Slope of the psychometric function. Conventions are the same as those in Fig. 2b. Also see Supplementary Fig. 1.

Due to possible leakage of the BS illumination outside the BS, the observers may have perceived the blue light. Therefore, to confirm that no such leakage had contributed to brightness perception, we conducted Experiment 2 (Fig. 3a). A bright red annulus surrounding the BS was presented simultaneously with both the test and reference arcs. The procedure was otherwise the same as that of Experiment 1—the reference arc was initially presented without BS illumination, followed by the test arc accompanied by BS illumination in half of the trials. Because the luminance of the red annulus (11.19 cd/m^2^) was much higher than that of the blue light of the BS illumination, the luminance outside the BS could only *decrease*, even if the BS stimulus had undergone misalignment (Fig. 3b). Even with this modification in Experiment 2, we still found that the test arc was judged as *darker* (*t*_8_ = −2.94, *p* = 0.0188) with the BS illumination than without it (Fig. 4a), whereas the slope of psychometric function was similar (*t*_8_ = −1.33, *p* = 0.222; Fig. 4b).

In Experiment 3, we presented the BS with a large blue-light stimulus whose outer radius (5 deg) was sufficiently larger than that of the BS (approximately 1.5 deg) to mimic possible misalignment and/or local scattering of the blue light within the optic disk used in Experiment 2 (Supplementary Fig. 1a). We found that the test stimulus appeared *brighter*, rather than darker, possibly because of the response bias when the observers perceived light around the BS and/or apparent motion across the hemifields. Nevertheless, this result is contrary to the findings of Experiment 2. To compare Experiments 3 and 2, we calculated the difference in PSE (‘ΔPSE’) between the ‘Illumination’ and ‘No illumination’ conditions for each observer (Supplementary Fig. 1b). We then performed a t-test between the ΔPSEs from Experiments 3 and 2, revealing that they differed significantly (*t*_10_ = 6.60, *p* < 0.0001). Furthermore, the ΔPSE in Experiment 3 was significantly greater than 0 (*t*_2_ = 6.28, *p* < 0.05 by two-tailed paired t-test with Bonferroni-correction) whereas the ΔPSE in Experiment 2 was less than 0 (t_8_ = −2.94, *p* < 0.05; Bonferroni-corrected). These results suggested that the shift in brightness in Experiment 2 was not due to the misaligned and/or scattered light received by a normal retinal region.

### Illumination inside the blind spot is perceptually undetectable

In Experiment 4, we delivered the BS illumination together with the reference arc in half of the trials, and together with the test arc in the remaining trials, and examined whether the observers could discriminate between these two conditions. In cases of perfect positional alignment, a uniformly filled red oval would be perceived in both conditions, because of perceptual filling-in induced by the red annulus. Indeed, the observers could not discriminate between these two conditions (*d*’ = 0.449 ± 0.469 [mean ± standard error], *t*_8_ = 0.956, *p* = 0.367). Thus, while the BS illumination influenced brightness perception elsewhere in the visual field—in the opposite hemifield, to be more precise—the BS illumination itself was not consciously accessible. The shift in brightness due to BS illumination was not correlated with *d*’ across observers (*r* = 0.166, *p* = 0.646), suggesting that the results of the brightness matching are unlikely to have been affected by inadvertent detection of the BS illumination due to misalignment.

### Flickering illumination inside the blind spot does not alter brightness

In Experiment 5, we examined whether the darkening of a remote stimulus by BS illumination was produced by a mechanism that could keep track of luminance modulation at a high temporal frequency. Two white stimuli (3.14 cd/m^2^) located in the second and third quadrants of the visual field were alternated at 10 Hz. The observers were asked to judge which of the two stimuli appeared brighter. In one condition, a white high-intensity rectangle (49.28 cd/m^2^) covering a large portion of the right hemifield was flickering at 10 Hz in synchronization with either the upper or lower stimulus (Supplementary Fig. 2a, left). In the other condition, we illuminated inside the BS with the same blue oval as used in Experiments 1 and 2 (3.01 cd/m^2^) but with flickering at 10 Hz (Supplementary Fig. 2a, right). We calculated the discriminability index (*d’*) for the in-phase and anti-phase stimuli; if the brightness-alteration effect involved a mechanism that could not resolve the flicker, the *d’* would be negligible. The results indicated that the observers could barely discriminate the in-phase and anti-phase stimuli with the rectangular flicker but never with the BS flicker (Friedman test, p = 0.0433) (Supplementary Fig. 2b). Moreover, three of four observers were able to discriminate the in-phase and anti-phase stimuli significantly better than chance with the rectangular flicker (chi-square test, p < 0.0019 for each of the three observers), whereas none of the observers could discriminate the in-phase and anti-phase stimuli with the BS flicker (chi-square test, p > 0.13 for each of the observers) (Supplementary Fig. 2c). These results indicated that the light projected onto a normal region of the retina affected brightness elsewhere via some mechanism that was sensitive to high temporal frequency; optical scatter is the most likely candidate mechanism. However, the mechanism sensitive to BS illumination was inadequate for resolving the 10 Hz flicker to establish synchronized brightness modulation, suggesting that optical scatter did not account for the darkening effect observed in Experiments 1 and 2.

### Brightness change correlates with PLR enhancement by blind-spot illumination

**Figure 5:**
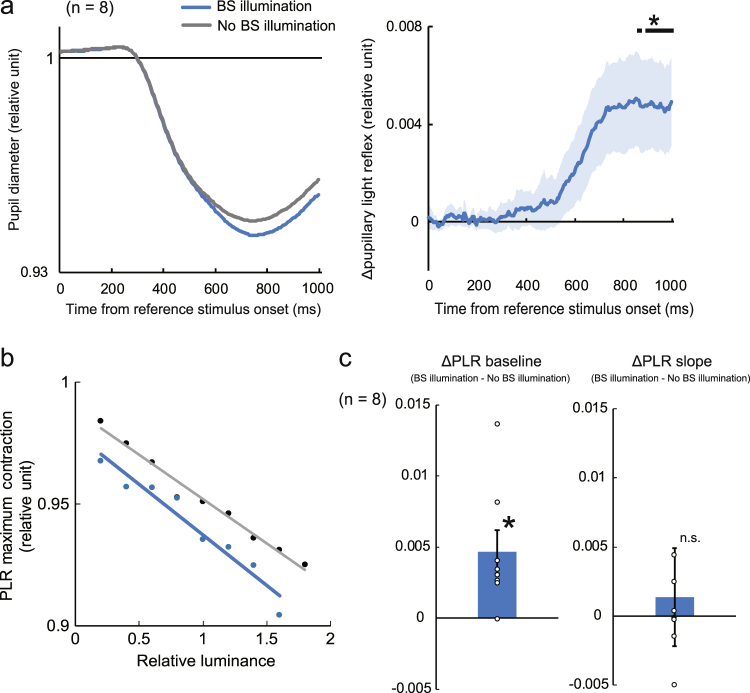
Relationship between brightness perception and enhanced pupillary light reflex (PLR) in the presence of illumination inside the blind spot (BS). (**a**) Left. Average time course (n = 8) of pupil diameter in response to the reference and test arcs with BS illumination (blue) and without BS illumination (black). Right. Average difference (n = 8; mean ± standard error) in pupil diameter between the conditions plotted as a function of time. *p < 0.05 (t-test against zero). (**b**) Amplitude of the maximum pupil contraction as a linear function of the luminance of the reference arc in a representative observer (relative to the test arc luminance, which was fixed at 3.14 cd/m^2^). (**c**) Differences between the midpoints (left) and between slopes (right) of the linear regression lines for the two conditions (mean ± standard error). *p < 0.05 (t-test against zero).

Finally, we examined whether short-latency PLR in response to the arc stimuli was enhanced by BS illumination applied simultaneously with the test arc. To this end, we separately analysed the pupil diameter data, which had been recorded during Experiment 1 with and without BS illumination. In accordance with our previous research, the amplitude of the PLR was greater when the test arc was presented with BS illumination than when it was presented alone (Fig. 5a). The latency of the enhancement (mean ± standard error: 519 ± 54.9 ms; Fig. 5a right) was longer than the latency of the initial contraction (Fig. 5a left), suggesting that the PLR is enhanced by a biological mechanism distinct from the rods/cones, and that this mechanism involves light reception within the BS. In each observer, the amount of PLR recorded during the brightness-matching experiment increased in proportion with the luminance of the reference arc (Fig. 5b), as predicted from previous studies^7^. In trials involving BS illumination, the PLR was generally enhanced, i.e., there was a significant difference between the midpoints of the linear regression lines for the two conditions (‘BS illumination’ and ‘No BS illumination’; *t*_7_ = 3.05, *p* = 0.0185; Fig. 5c, left); and conversely, the slopes did not differ significantly (*t*_7_ = 0.383, *p* = 0.713; Fig. 5c, right). These findings indicated that the BS illumination-mediated enhancement of PLR to the test arc acts substantially to the conventional PLR.

## Discussion

In summary, we found that blue light illumination inside the BS is invisible, but reduces the brightness of a white light outside the BS (Figs 2 and 4). One possible explanation for this phenomenon is that the blue light was scattered out of the BS and detected by rods/cones nearby. Indeed, myelinated axons within the optic disk have a higher reflectance than the pigment epithelium. Thus, the optic disk may scatter light back into the eyeball, in addition to diffusion of incident light within the vitreous humour. However, this light-scattering hypothesis is unlikely for the following three reasons. First, if light projecting onto the optic disk were scattered by 100% and uniformly spread over a circular area with a radius of 15 deg, the scatter would have led to an effect that was comparable to a 0.03 cd/m^2^ increase in background luminance; such a small difference would not explain the observed shift in brightness. Second, even if the light projecting onto the optic disk were spread within a smaller retinal area—e.g., twice as large as the estimated optic disk—the stimulation would have been ineffective, at least in Experiment 2, because the border of the BS would have been masked by the much brighter, red annulus stimulus (see Fig. 3). Third, when the small blue light inside the BS was replaced by a large blue light covering an area outside the BS, apparent brightening rather than darkening occurred (Supplementary Fig. 1). This stimulation mimicked local scatter, but diffusive scatter was also included since the stimulation occurred through the ocular media. If either local or diffusive scattering had a role in the apparent darkening, the same darkening effect is expected. Thus, it is unlikely that reduced brightness perception by illumination inside the BS observed in Experiments 1 and 2 was caused by scattering.

**Figure 6:**
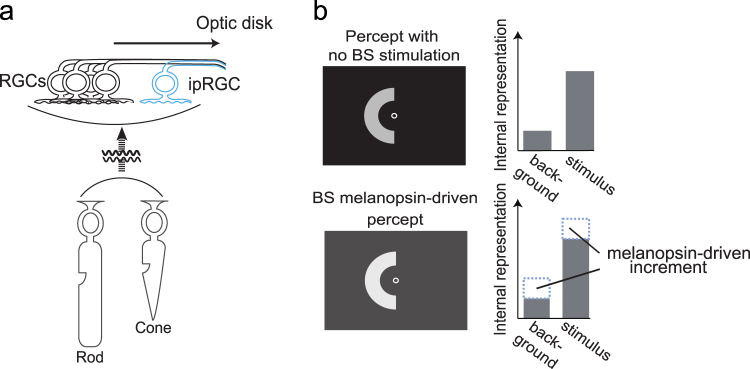
Proposed mechanism of the darkening effect. (**a**) Schematic view of ipRGCs in the retina. The axons of conventional RGCs and ipRGCs converge into the optic disk. Each ipRGC contains melanopsin within its soma and axon. (**b**) Schematic of the proposed mechanism for the BS illumination-mediated darkening effect of a remote stimulus. Because the luminances of both the stimulus and its background are interpreted as greater, the ratio of the interpreted luminance of the stimulus to that of its background becomes smaller.

We propose that the collective results could be explained based on the key role of a photopigment, melanopsin^9^ (Fig. 6a). Melanopsin has been found in intrinsically photosensitive retinal ganglion cells (ipRGCs), whose activation is considered essential for ‘non-image-forming vision’, such as the PLR^10^, as well as for the regulation of the circadian rhythm^11^. Anatomical studies have identified immunostained melanopsin not only in the ipRGC somas, but also along the plasma membranes in the optic-fibre and ganglion-cell layers^11^. Axons containing melanopsin have been reported to extend across the retinal surface and converging at the optic disk, indicating that melanopsin is expressed wherever incoming light strikes the retina (both inside and outside the BS), and not after the optic nerve leaves the retina and enters the brain. We propose that melanopsin activation at the optic disk impacts visual processing in ipRGCs. It is known that the somas of ipRGCs are located outside the BS, receive light on their own, and also process visual signals originating from the rods/cones^10,12–14^. Excitation of the ipRGC axons in the optic disk may contribute to brightness perception via neural pathways of the ‘image-forming vision,’ in addition to contribution to non-image-forming vision, as reported in our previous study related to PLR enhancement^8^. Moreover, melanopsin has peak light absorption at 482 nm^15^, indicating that it is most responsive to blue light^16^. Meanwhile, the rods and S-cones have peak light absorption at around 498 nm and 420 nm, respectively^17^. Thus, photoreceptors such as rods and S-cones along the border of the BS may cause the stimulus outside the BS to appear darker. However, this scenario is unlikely because we confirmed that deliberately stimulating nearby rods/cones did not induce the same effect but rather caused the stimulus to appear brighter (Supplementary Fig. 1). Moreover, when we illuminated inside the BS with blue light flickering at 10 Hz, no evidence of brightness modulation was found, suggestive of the possible involvement of melanopsin (Supplementary Fig. 2). This result is consistent with the report that melanopsin-based visual responses are too sluggish to be sensitive to luminance modulation at frequencies higher than 4 Hz^18^. These collective observations point to a biological mechanism that is distinct from conventional photoreceptors and specifically responsive to light inside the BS.

The remaining question is why illumination inside the BS should make another remote stimulus *darker*. We propose that BS illumination uniformly influences perception within the entire visual field outside the BS. In our previous report^8^, BS illumination cannot by itself trigger the PLR but is able to enhance the amount of PLR activated by abrupt uniform increment of luminance outside the BS. Thus, BS illumination facilitates ocular ‘photometry’ of the entire visual field. If the same biological mechanism applies to image-forming vision, it may be that the system considers the whole visual field to have higher luminance with BS illumination, as compared to that with no BS illumination (Fig. 6b). In an internal representation, not only the light stimulus outside the BS but also the background will be interpreted to have a higher luminance. Therefore, the ratio of interpreted target luminance to background luminance will become smaller, and in keeping with classical Fechnerian scaling, the stimulus will appear *darker*, as compared to that under the condition of no BS illumination.

Our study revealed that the rod/cone-free optic disk, which has been believed to be a totally ‘blind’ spot, actually serves visual functions that influence our perception. Furthermore, melanopsin in the retina outside the BS, presumably expressed in the cell bodies of the ipRGCs, may contribute to image-forming vision^18–21^. Several studies have reported that each ipRGC can also encode spatiotemporal information at low spatial and temporal frequencies^22,23^. However, it is unknown how melanopsin expressed along the axons of the ipRGCs might contribute to our visual experience. The present study suggested that the BS serves as a reference for environmental illumination in the brightness scaling of objects around us, and that it does so by influencing image-forming vision via ipRGC excitability.

## Methods

### General methods

This study conformed to the Declaration of Helsinki guidelines and was approved by the institutional ethics committee of the Graduate School of Humanities and Sociology, University of Tokyo. The methods were carried out in accordance with the approved guidelines. Informed consent was obtained from all observers. Seventeen healthy adults who were naïve to the purpose of the experiment, and three of the authors (MS, KM, and YU) participated (21–32 years old, 5 females and 15 males). All observers had normal or corrected-to-normal visual acuity. In each observer, 18–36 trials were carried out at each point constituting a psychometric function.

Experiments were conducted in a dark room. All stimuli were displayed on an LCD monitor (VIEWPixx/3D Lite; VPixx Technologies; 1.5 arcmin/pixel, 49.0 deg × 31.7 deg) with a refresh rate of 120 Hz under the control of a computer (Apple Mac Pro). As a programming environment, we used Matlab (MathWorks), with the Psychophysics Toolbox^24–26^, Eyelink Toolbox^27^, and Palamedes Toolbox^28^ extensions. Each observer’s head was constrained using a chin rest. The viewing distance was fixed at 52 cm. The left eye was completely occluded using an opaque eye patch (CoMo Good non-pressure black type). The pupil diameter and gaze position of the right eye were always recorded with an eye tracker (SR Research Eyelink II) at a sampling rate of 250 Hz.

Prior to the main experiments, the blind spot (BS) of each observer’s right eye was mapped using the method reported previously^8,29^. First, an oval stimulus was presented to the right of a fixation point located at the centre of the screen. Each observer then adjusted the location and size of the oval using a computer mouse and keys. Next, the oval stimulus was turned off, and a small spotlight (24.5 arcmin in diameter) was displayed near the oval border. The observers pressed a key as soon as they detected the spot. Fifty-six spots (combinations of seven distances from the border and eight radial directions from the centre of the oval) were displayed in random order during each session. This spotlight perimetry around the border was repeated for at least eight sessions. The position that corresponded to a 50% detection rate was taken as the detection threshold in each radial direction, and adjacent points were connected by straight lines to create a polygon. The largest oval that fitted just inside the polygon was determined geometrically; this oval was then further shrunk to 90% of its original size to avoid unintended misalignment due to fluctuations in gaze fixation. In this way, BS illumination in the brightness-matching experiment was confined within the actual BS.

### Procedure of Experiment 1: brightness matching with and without the illumination of the blind spot

Observers (*n* = 10) performed a two-alternative forced choice (2AFC) task for brightness judgement (Fig. 1). We used two white stimuli (CIE [*x*, *y*] = [0.322, 0.357] as measured using a spectroradiometer [Konica Minolta CS-2000]), namely the reference and test arcs. The reference arc was a semicircle covering the right perifovea (eccentricity: 5–10 deg); the test arc was a semicircle covering the left perifovea; the reference and test arcs were symmetrical about the vertical meridian. The test arc was confined within the left hemifield to prevent any physical interaction with the simultaneous illumination in the right-eye BS, which is located in the right hemifield. Each trial began after the observers had maintained fixation to within ±0.955 deg about the central fixation point for at least 500 ms.

First, the reference arc was presented for 50 ms in the right hemifield. After an inter-stimulus interval of 200 ms, the test arc was presented for 50 ms in the left hemifield. In half of the trials, the BS, which had been determined using the above procedure, was illuminated by blue light (3.01 cd/m^2^; CIE [*x*, *y*] = [0.138, 0.0501]). The spectral distribution was restricted using a band-pass filter (Fuji Film BPB-45; wavelength peak: 462 nm; full width at half maximum: 19 nm) for 100 ms. Its onset was 50 ms prior to the test arc onset, while its offset was synchronized with the test arc offset. A beep was delivered 800 ms after the stimulus presentation, and the observers reported whether the test arc appeared brighter or darker than the reference arc by pressing one of two computer keys. An inter-trial interval was randomly chosen within a range of 2.5 ± 0.5 s.

The luminance of the test arc was fixed at 3.14 cd/m^2^, whereas the reference arc had various levels of luminance predetermined for the method of constant stimuli. In four out of the 10 observers, both the test and reference arcs were accompanied by another blue oval as large as the BS. This oval was located to the right of the BS by 1.5× the BS width; its onset was 50 ms prior to the arc onset, and its offset was synchronized with the arc offset. It was used as a mask to discourage observers from searching for hints in the BS illumination. We confirmed that the presence or absence of the mask did not affect behavioural results.

### Procedure of Experiment 2: brightness matching with a red annulus surrounding the blind spot

To confirm that the rods/cones outside the BS had not contributed to the behavioural data, we conducted Experiment 2 in 11 observers. The only procedural difference from Experiment 1 was the presence of a red annulus (CIE [*x*, *y*] = [0.681, 0.303]; Fig. 2) surrounding the BS. This annulus had the same area as the blue BS illumination; as such, the inner and outer perimeters of the annulus were 100% and 141%, respectively, as large as the estimated BS border. The annulus was presented 50 ms before the test and reference arcs, regardless of whether BS illumination was presented, and was extinguished together with the arc offset. The luminance of the red annulus (11.19 cd/m^2^) was much higher than that of the blue BS illumination (3.01 cd/m^2^).

### Procedure of Experiment 3: simulated local scatter

We mimicked the possible scattering of the blue light projected inside the BS (observers n = 3, all of whom also participated in Experiment 2; Supplementary Fig. 1). The sequence of each trial was the same as in Experiment 2. In half of the trials, instead of actual BS illumination, but following the same time course, we presented a large blue-light stimulus (0.56 cd/m^2^) centred at the BS centre. This stimulus had an annular shape with its inner perimeter overlapping the estimated BS border and its outer perimeter with a radius of 5 deg. The total energy of blue light illumination *outside* the BS in this experiment was set as almost equivalent to that *inside* the BS in Experiment 2.

### Procedure of Experiment 4: detectability of the illumination inside the blind spot

To confirm that the observers in Experiment 2 could not consciously detect the BS illumination, they were asked to perform a detection task (*n* = 11). The sequence of each trial was the same as in Experiment 2. However, in every trial, the BS illumination was delivered together with either the reference or test arc. The observers were asked to judge, in a 2AFC paradigm, which arc was accompanied by BS illumination. We collected data from 84 trials and carried out a signal-detection-theory analysis.

### Procedure of Experiment 5: brightness modulation by flickering illumination inside or outside the blind spot

Observers (*n* = 4) performed a 2AFC task for brightness judgement of flickering stimuli (Supplementary Fig. 2a). Two white arc stimuli (3.14 cd/m^2^; CIE [x, y] = [0.322, 0.357]) located in the second and third quadrants of the visual field were turned on and off with a duty cycle of 1:1 at 10 Hz, antiphase to each other. The total duration was 300 ms. The upper and lower arcs subtended 10–12 o’clock and 6–8 o’clock, respectively (eccentricity: 5–10 deg). In one condition, a white rectangle (49.28 cd/m^2^; CIE [x, y] = [0.3056, 0.3360]), subtending 5–24.5 deg horizontally and ±15.85 deg vertically with respect to the fovea, was flickering with a duty cycle of 1:1 at 10 Hz in phase with either the upper or lower arc with equal probability, for the 300 ms duration. In the other condition, the rectangle was replaced by the same blue oval inside the BS as used in Experiments 1 and 2 (3.01 cd/m^2^; CIE [x, y] = [0.138, 0.0501]), flickering in phase with either arc. The observers were asked to judge which arc appeared brighter. Responses were collected for 200–240 trials for each condition.

### Quantification and statistical analysis

We obtained a psychometric function by fitting the proportion of the reference arc that was judged as brighter with the cumulative binomial function. The midpoint of the best-fit function was taken as the point of subjective equality (PSE) and the slope around the PSE as an index of sensitivity. Trials that included blinks or large eye movements (>1 deg) during stimulus presentation were removed from the analysis. Observers were excluded from the analysis as poor performers if their ordinate proportion values corresponding to the highest and lowest abscissa values differed by <0.6 in the psychometric function in the ‘No BS illumination’ condition (one of the nine observers in Experiment 1, and two out of the 11 observers in Experiments 2 and 4). However, when we included these observers in the analysis, our main conclusions did not change.

In an off-line analysis, the pupil diameter in each trial was normalised as a fraction of the baseline diameter averaged over the 500 ms before stimulus onset. Trials that included blinks within 1 s of the stimulus onset were removed. For each combination of stimulus conditions (nine reference arc luminances × two BS illumination conditions [with and without]), we averaged the pupil diameter time course across all trials within observer to identify the time at which the pupil diameter became minimal. Next, we plotted the PLR amplitude at maximum contraction against reference arc luminance, for each observer and for both ‘BS illumination’ and ‘No BS illumination’ conditions (see Fig. 5b). As predicted from previous studies^7^, the size of PLR increased linearly—or the pupil diameter decreased linearly—with reference arc luminance. We confirmed that there was no significant interaction between BS illumination and reference arc luminance. Therefore, we fitted the data with a linear regression line and took its midpoint—where reference arc luminance was equivalent to test arc luminance—as an index of the PLR size for each of the BS illumination conditions. We also calculated, in each observer, the second-order derivative (acceleration) of the averaged pupil diameter as a function of time. The time from stimulus onset to minimum (i.e., the most negative) acceleration was taken as the PLR latency.

### Data Software Availability

The data and code that support the findings of this study are available from the corresponding author upon reasonable request.

## Electronic supplementary material

Supplementary information
