# Supplementary material for: Invisible light inside the natural blind spot alters brightness at a remote location
Source: Sci Rep. 2018 May 15;8:7540. doi: 10.1038/s41598-018-25920-9 (PMC5954096; doi:10.1038/s41598-018-25920-9)
Supplement: Supplementary file 1 — Supplementary information [file 41598_2018_25920_MOESM1_ESM.pdf]

Supplementary information

Invisible light inside the natural blind spot alters brightness at a remote location

Marina Saito[1,5], Kentaro Miyamoto[2,3,4,5], Yusuke Uchiyama[1], Ikuya Murakami[1*]

1 Department of Psychology, The University of Tokyo, Tokyo 113-0033, Japan.

2 Department of Physiology, The University of Tokyo School of Medicine, Tokyo 113-0033, Japan.

3 Department of Experimental Psychology, University of Oxford, Oxford OX1 3UD, United Kingdom.

4 Japan Society for the Promotion of Science, Tokyo 102-8472, Japan.

5 These authors equally contributed.

*Correspondence and requests for materials should be addressed to I.M. (ikuya@l.u-tokyo.ac.jp)

Supplementary Figures

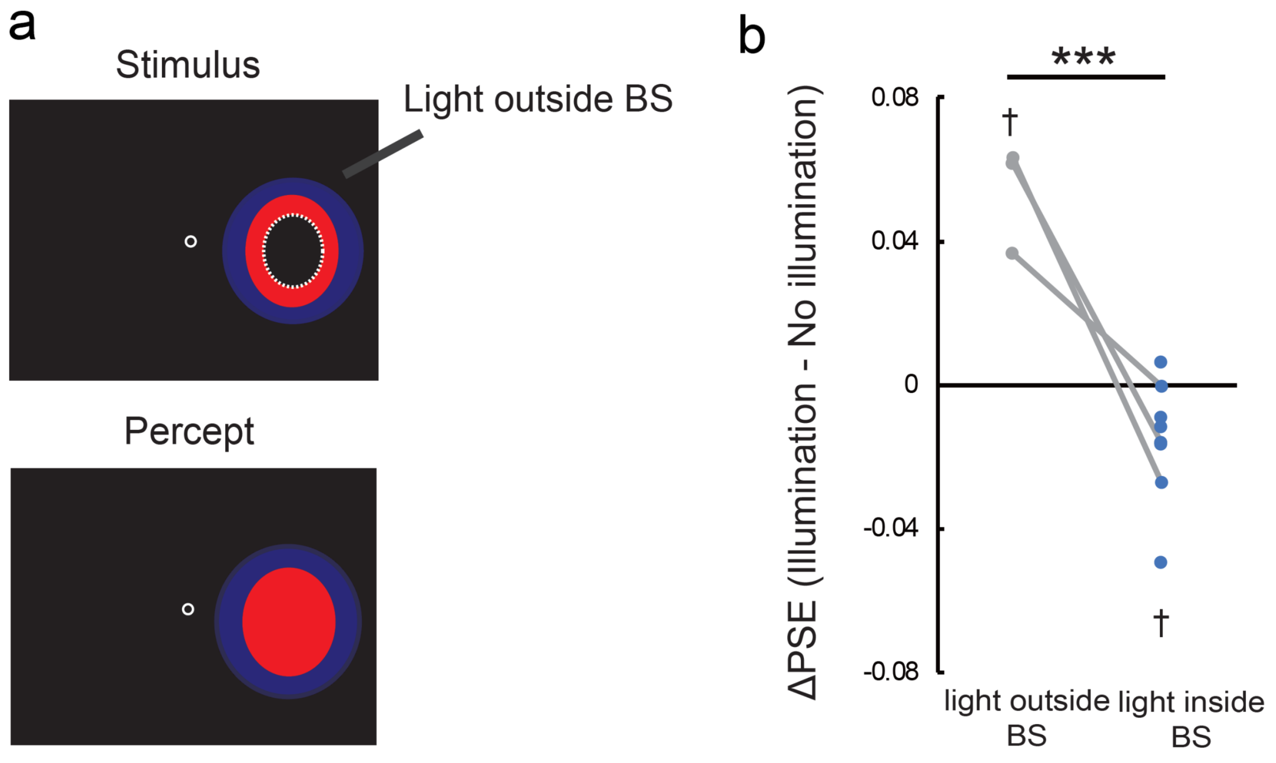

Supplementary Figure 1. Stimuli and results of Experiment 3 demonstrating brightness increase by illumination with blue light larger than the blind spot (BS) tested as a simulation of local scatter

a. Schematic of the stimulus configuration in Experiment 3. A red annulus surrounded the BS as in Experiment 2, but blue-light illumination was presented within an annular region surrounding the red annulus, such that the blue light was available to the conventional photoreceptors on the retina outside the BS. The sequence of each trial was the same as in Experiment 2.

b. Comparison of PSE in Experiment 3 with that in Experiment 2. The ordinate ('ΔPSE') indicates the difference in PSE between the 'Illumination' and 'No illumination' conditions; thus, a greater ΔPSE indicates that the test arc was brighter than the reference arc. The 'light outside BS' and 'light inside BS' conditions correspond to the data of Experiments 3 and 2, respectively. Each point corresponds to each observer's ΔPSE. ***$p < 0.001$ (unpaired t-test); †$p < 0.05$ (Bonferroni-corrected, t-test against zero).

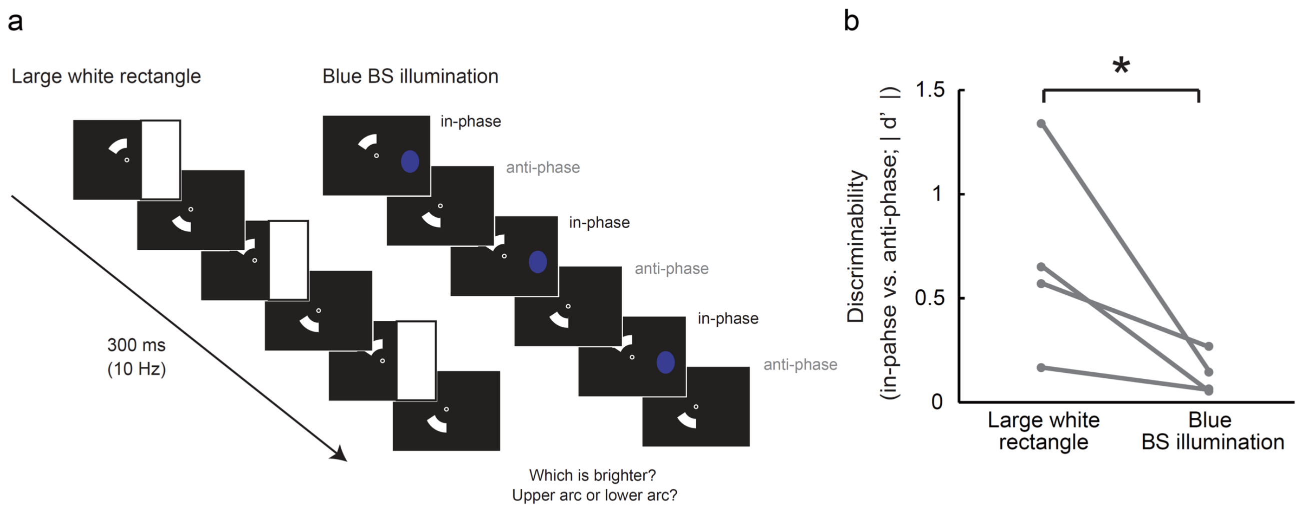

c

	Large white rectangle		Blue BS illumination	
Observer	Cramer's V	p value	Cramer's V	p value
O1	**0.488**	**<0.001*****	0.054	0.407
O2	**0.251**	**<0.001*****	0.021	0.750
O3	0.066	0.363	0.025	0.726
O4	**0.218**	**0.001****	0.099	0.136

Supplementary Figure 2. Stimuli and results of Experiment 5 demonstrating that flickering illumination inside the blind spot does not alter brightness

a. Schematics of the stimulus configurations in Experiment 5. Two arcs were alternated at 10 Hz. A large white rectangle (left) or blue BS illumination (right) was flickering in synchronization with the arcs.

b. Interobserver comparison of discriminability index (|d'|) between the rectangle and BS illumination. * $p < 0.05$ (Friedman test).

c. Intraobserver evaluation of the discriminability between the in-phase and anti-phase arcs by χ^2 test (Cramer's V $= \sqrt{\chi^2/N}$ where N denotes sample size). No observers could significantly discriminate the in-phase and anti-phase arcs with the BS flicker by blue light (chi-square test, $p > 0.13$ for each). The discriminability was not improved even if the BS illumination was not made with the blue light but with white high-intensity light (49.28 cd/m^2) (chi-square test, $p > 0.36$ for each observer).
